# Chromosome-level genome assembly of the pygmy grasshopper *Eucriotettix oculatus* (Orthoptera: Tetrigoidea)

**DOI:** 10.1038/s41597-024-03276-2

**Published:** 2024-04-26

**Authors:** Ran Li, Yingcan Qin, Wantao Rong, Wei-an Deng, Xiaodong Li

**Affiliations:** 1 Guangxi Key Laboratory of Sericulture Ecology and Applied Intelligent Technology, Hechi University, Hechi, China; 2 College of Life Sciences, Qufu Normal University, Qufu, China; 3 School of Chemistry and Bioengineering, Hechi University, Yizhou, China; 4 College of Life Sciences, Guangxi Normal University, Guilin, China

**Keywords:** Chromosomes, Genome

## Abstract

The pygmy grasshoppers, which belong to the superfamily Tetrigoidea, exhibit remarkable environmental adaptability. However, no study has yet reported a reference genome for this group. In this study, we assembled a high-quality chromosome-scale genome of *Eucriotettix oculatus*, which survive in the environment heavily polluted by heavy metals, achieved through Illumina and PacBio sequencing, alongside chromosome conformation capture techniques. The resulting genome spans 985.45 Mb across seven chromosomes (range: 71.55 to 266.65 Mb) and features an N50 length of 123.82 Mb. Chr5 is considered to be the single sex chromosome (X). This genome is composed of 46.42% repetitive elements and contains 14,906 predicted protein-coding genes, 91.63% of which are functionally annotated. Decoding the *E. oculatus* genome not only promotes future studies on environmental adaptation for the pygmy grasshopper, but also provides valuable resources for in-depth investigation on phylogeny, evolution, and behavior of Orthoptera.

## Background & Summary

The pygmy grasshoppers of the superfamily Tetrigoidea (Orthoptera) constitute a single cosmopolitan family, Tetrigidae, which is widely distributed throughout the world and has 287 genera within seven subfamilies^1–3^. The species generally inhabit moist environments such as mountain streams, small rivers, swamps, grasslands and bushes, feed on humus, mosses, and lichen^1,4^. Their distribution is highly dependent on specific natural environments, especially some are very sensitive to microhabitat changes, hence, they are important environmental indicator species^5,6^. Meanwhile, the pygmy grasshoppers have complex behaviors and are generally regarded as ideal materials for behavioral researches^1,7^. Some tetrigoid species have shown the ability to survive in environments contaminated with heavy metals^8^. However, studies of this family mainly focused on morphology, biology and ecology for the past decades, with a few studies on molecular mechanism of ecological and biological characteristics^9–12^. The lack of genomic information has made it difficult to conduct in-depth investigations of the pygmy grasshoppers.

**Fig. 1:**
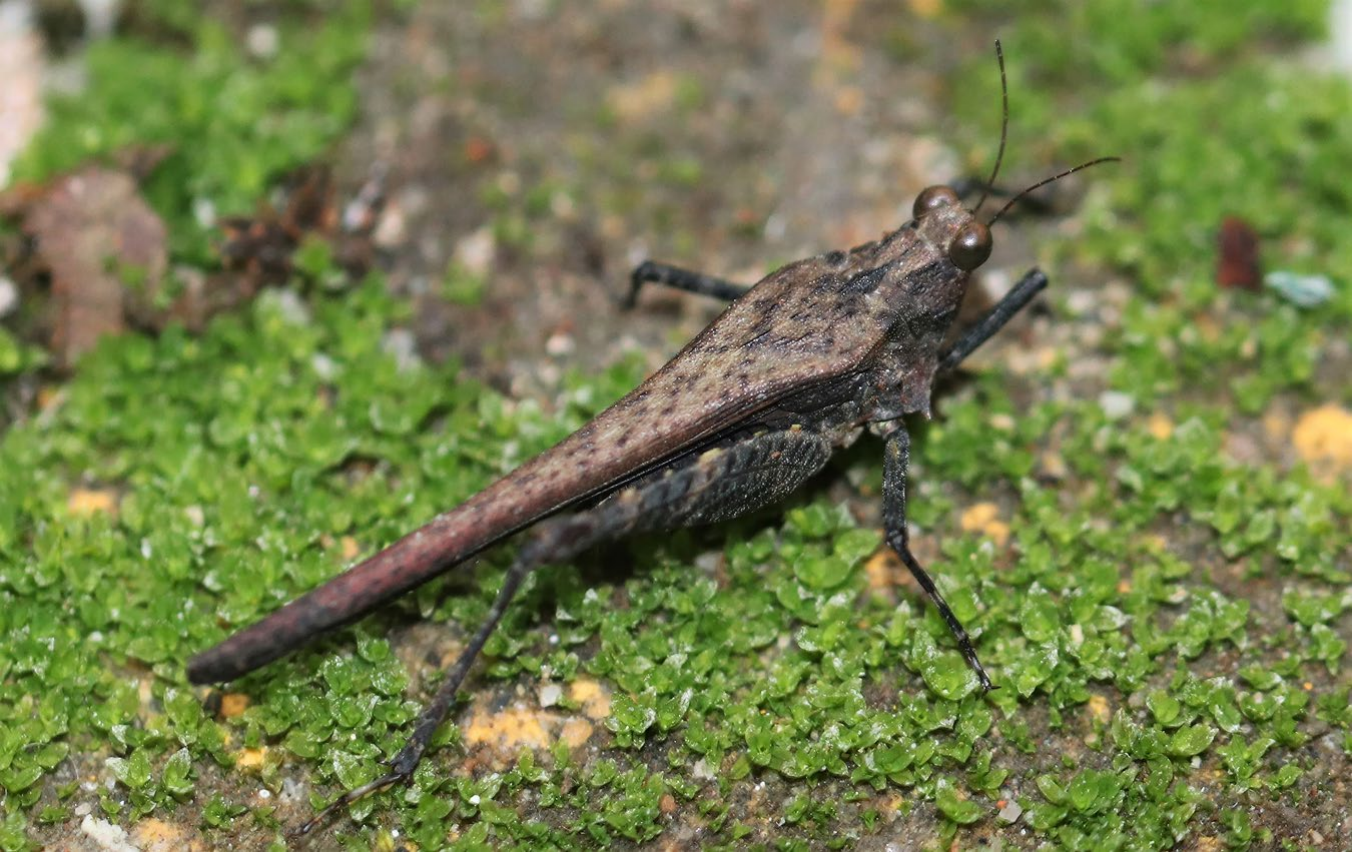
Habitus of *E. oculatus*.

*Eucriotettix oculatus* (Bolivar, 1898) is a typical Oriental species belonging to the genus *Eucriotettix* in the family, widely distributed in the southern provinces of China and South Asia region (Fig. 1)^13^. This species has strong adaptability to different environments, and the population which lives in mining regions around the Diaojiang River (China) has been polluted for hundreds of years^14^. Our previous analysis showed the composition and diversity of the intestinal microbial community of *E. oculatus* was significantly reduced in heavy metal pollution^15^. Meanwhile, we also found that heavy metals could change the composition of metabolites in the intestine^16^. However, there is limited knowledge on molecular mechanisms that support the environmental adaptation of *E. oculatus* to heavy metal pollution due to the gaps in genomic information.

In the present study, we reported the first genome of a pygmy grasshopper in the superfamily Tetrigoidea including the determination of the X chromosome. The high-quality genome was *de novo* assembled using integrated technologies (Illumina sequencing, PacBio sequencing, as well as proximity ligation chromatin conformation capture) to assist in chromosome-level assembly. We successfully annotated the protein-coding genes (PCGs), repetitive elements (REs), and non-coding RNAs (ncRNAs) within the genome. This high-quality genome will be a valuable resource for in-depth studies on basic biological possesses and environmental adaptation of the pygmy grasshopper.

## Methods

### Animal materials

Specimens of *E. oculatus* were originally collected from a wild population in Yizhou, Guangxi, China, and subsequently maintained at Hechi University for further study. Only adult speciments were utilized for high-quality genomic DNA and RNA extraction. The female bodies were collected for Illumina and PacBio genome sequencing, and muscle tissues of legs were prepared for transcriptome and Hi-C sequencing.

### Genome and transcriptome sequencing

Five female specimens were pooled and total DNA was then extracted using a Blood & Cell Culture DNA Mini Kit (Qiagen). DNA quantity and quality were finally measured by a 2100 Bioanalyzer (Agilent) and a Qubit 3.0 Fluorometer (Invitrogen), with integrity confirmed via 1% agarose gel electrophoresis. Whole-genome shotgun sequencing was performed for five female individuals with a single molecule real-time (SMRT) PacBio system. PacBio Sequel II libraries (insert size of 30 kb) were constructed with SMRTbell^TM^ Template Prep Kit 2.0. Additionally, two short paired-end libraries were prepared with Truseq DNA PCR-free kit, and short reads were yielded on the Illumina NovaSeq 6000 platform.

Muscle tissues of five female insects were collected for constructing pseudo-chromosomes. The Hi-C library was constructed according to the standard protocols described previously^17^. After quality control, 150 bp paired-end reads (PE150) were also generated by the Illumina NovaSeq 6000 platform. RNA of five female and male individuals (three biological replicates) was isolated using TRIzol Total RNA Isolation Kit (Takara). The cDNA library was built using TruSeq RNA Sample Prep Kit v2 (Illumina) and sequenced on the Illumina HiSeq 6000 platform using the paired-end strategy.

### Genome size estimation

**Fig. 2:**
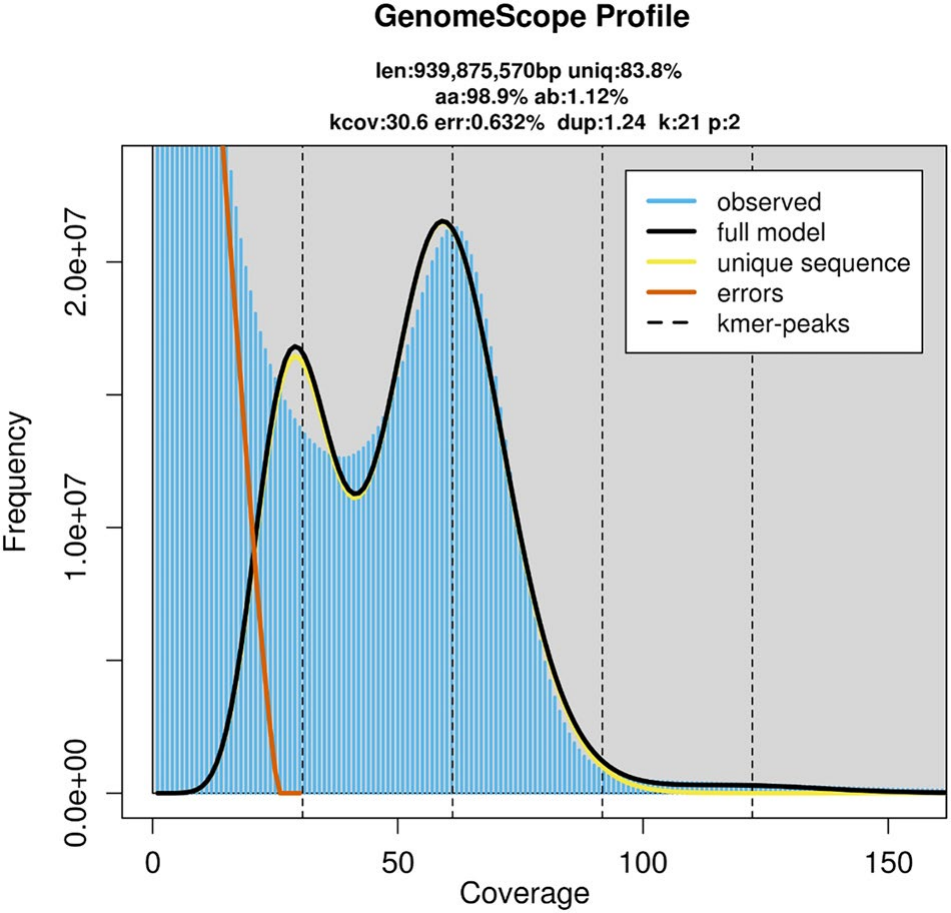
K-mer distribution of Illumina paired-end reads using GenomeScope based on a k value of 21.

In order to get a preliminary understanding of the genome size and other genome characteristics, a total of 97.53 Gb Illumina reads of female individuals were firstly produced (Table 1). Quality control was performed using the BBTools v38.82 package^18^. The 21-mer distribution was calculated using “khist.sh” (BBTools), and the genome survey analysis was carried out using GenomeScope v2.0^19,20^. Based on the k-mer distribution of the cleaned data, the genome size was fell within the range of 936.54–939.87 Mb (Fig. 2, Table 2). The genome size was determined to be 939.87 Mb with the number of unique k-mers peaked at 21.

**Table 1:** Statistics of the DNA sequence data used for genome assembly.

Illumina (Female)	350	650,174,472	97,526,170,800	150	/
Illumina (Male)	350	131,475,776	39,442,732,800	150	/
PacBio	30,000	5,957,992	85,763,544,541	14,394.71	25.168
Hi-C	350	380,773,738	114,232,121,400	150	/
RNA-seq	350	289,583,508	43,437,526,200	150	/

**Table 2:** The information of genome survey analysis.

Property	Minimum	Maximum
Homozygous (aa)	98.8589%	98.9001%
Heterozygous (ab)	1.09993%	1.14114%
Genome haploid length	936,544,462 bp	939,875,570 bp
Genome repeat length	151,511,721 bp	152,050,619 bp
Genome unique length	785,032,741 bp	787,824,951 bp
Model Fit	78.9684%	91.7688%
Read Error Rate	0.631679%	0.631679%

### Genome assembly

**Fig. 3:**
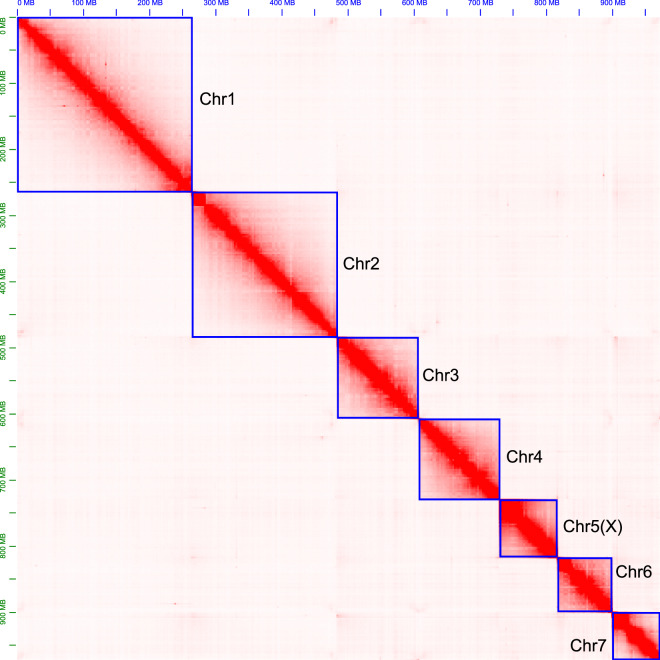
Hi-C contact heatmap of the *E. oculatus* genome.

A total of 85.76 Gb PacBio long reads (~91.3-fold coverage of the estimated genome size) were obtained after removing adaptors in polymerase reads with default parameters. The mean length and N50 length of PacBio subreads were 14.39 and 25.17 kb, respectively (Table 1). After self-corrected and long read polished, genome initial assembly was performed using the Flye v2.7.1^21^. As a result, we generated a 1.06 Gb genome assembly with the contig N50 of 1.95 Mb (Table 3). The size of the primary assembled genome was significantly larger than the genome size estimated by k-mer analysis. To further improve the quality and accuracy, we corrected the genome by removing haplotigs and contig overlaps from the genome, and short-read polishing with high coverage of Illumina reads using Purge dups v1.0.1 and NextPolish v1.1.0, respectively^22,23^. Total size of the draft genome assembly was 993.58 Mb with an N50 length of 2.3 Mb (Table 3). To produce the chromosome-level assembly, 114.23 Gb Hi-C sequencing data (380,773,738 reads) was generated and used to anchor contigs into pseudo-chromosomes with 3D-DNA v180922 pipeline^24^. Juicebox v1.6.2 was subsequently employed to review and manually curate scaffolding errors^25^. Finally, a high-quality chromosome-level genome assembly was generated after JBAT review. Approximately 279 million unique mapped reads (73.40%) and 126 million valid reads (33.10%) were produced. 973.09 Mb data on the base level was anchored and orientated onto 7 chromosomes with a mounting rate of up to 98.78%, and the chromosome lengths ranged from 71.55 to 266.65 Mb (Table 4). After scaffolds were clustered, ordered and orientated to restore their relative locations, the heatmap of chromosome crosstalk indicated that the genome assembly was robust and complete (Fig. 3). Finally, the size of this genome was 985.45 Mb, consisting of 248 scaffolds and 1,944 contigs with an N50 length of 123.82 and 2.09 Mb, respectively (Table 5). Results showed that the size of the assembled pygmy grasshopper genome is close to the genome the estimated size, suggesting that the non-redundant genome was appropriate.

**Table 3:** Summary of each step in construction of the *E. oculatus* genome assembly.

Assembly	Total length (Mb)	Number of scaffolds (chromosome)	N50 length (Mb)	Longest scaffold (Mb)	GC (%)	BUSCO (n = 1,367) (%)			
						C	D	F	M
Flye	1,058.845	8,778	1.951	27.808	35.15	97.1	1.6	0.5	2.4
Purge Dups	997.307	3,783	2.285	27.808	35.06	97.3	1.1	0.5	2.2
NextPolish	993.578	3,782	2.3	27.729	35.09	97.3	1.2	0.6	2.1
Hi-C	985.445	248 (7)	123.817	266.651	35.08	97.4	0.9	0.6	2.0
Final assembly	985.445	248 (7)	123.817	266.651	35.08	97.4	0.9	0.6	2.0

**Table 4:** Statistics of chromosome-level genome assembly of *E. oculatus*.

Chr ID	Length (bp)	Average sequencing depth (X)
Chr1	266,650,797	38.1563
Chr2	219,134,839	37.9575
Chr3	123,817,100	36.6892
Chr4	120,453,506	36.4045
Chr5 (X)	89,326,600	**19.5598**
Chr6	82,152,429	36.9717
Chr7	71,551,646	38.3184

**Table 5:** Genome assembly and annotation statistics of *E. oculatus*.

Elements	Value
*Genome assembly*	
Assembly size (Mb)	985.445
Number of scaffolds/contigs	248/1,944
Longest scaffold/contig (Mb)	266.651/16.579
N50 scaffold/contig length (Mb)	123.817/2.093
GC (%)	35.08
Gaps (%)	0.017
BUSCO completeness (%)	97.4
*Gene annotation*	
Protein-coding genes	14,906
Mean protein length (aa)	522.08
Mean gene length (bp)	15352.03
Exons/introns per gene	9.45/8.19
Exon (%)	4.14
Mean exon length	288.38
Intron (%)	19.08
Mean intron length	1542.64
BUSCO completeness (%)	95.2

### Sex chromosome determination

To identify the X chromosome of *E. oculatus*, resequencing for males produced a total of 39.4 Gb high-quality data with a mean Q30 of 93.5% (Table 1). The data was then mapped to 7 chromosomes, and the sequencing depth was used to identify the X chromosome. The results showed that the mean sequencing depth of Chr1-4, 6 and 7 was nearly two-fold greater than that of Chr5. The Chr5 was hence considered to be the X chromosome (Fig. 3, Table 4).

### Repeat annotation

**Fig. 4:**
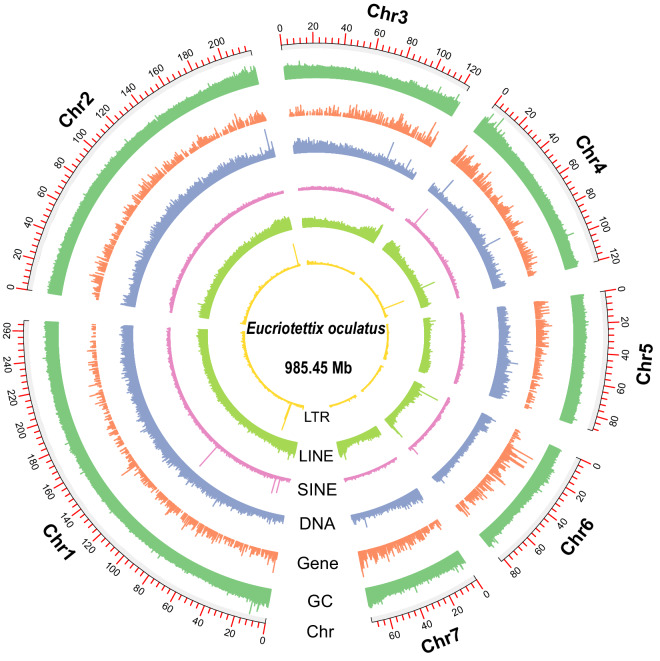
Schematic representation of the genomic characteristics of *E. oculatus*. The outer rings of the circle represent the distribution of long terminal repeats (LTRs), long interspersed nuclear elements (LINEs), short interspersed nuclear elements (SINEs), DNA elements, genes, GC content, and chromosomes.

Repetitive elements (REs) were detected by two routine approaches, including ab initio and homology prediction. For ab initio prediction, RepeatModeler v2.0.1 was firstly used to identify the REs, and a *de novo* repeat sequence library was subsequently built using the results^26^. Finally, a custom library was constructed combining with two databases (Dfam v3.1^27^ and RepBase v20181026^28^). For homology prediction, REs were masked by RepeatMasker v4.1.0 on the custom library^29^. A total of 457.39 Mb REs were identified (constituting 46.42% of *E. oculatus* genome), including 45.03% transposable elements (TEs), 1.01% simple repeats, 0.16% low-complexity regions, and 0.15% small RNAs, 0.06% satellites (Fig. 4). The predominant 6 categories of TEs were unclassified (19.47%), long interspersed nuclear elements (LINEs, 15.61%), DNA transposon elements (5.78%), rolling-circles (RCs, 2.42%), long terminal repeats (LTRs, 1.06%), and short interspersed nuclear elements (SINEs, 0.69%).

All ncRNAs (rRNAs, snRNAs and miRNAs) were detected by Infernal v1.1.3^30^ and tRNAscan-SE v2.0.7^31^, yielding 5,514 tRNAs (21 isotypes, Supres lacking), 37 small nuclear RNAs (snRNAs), 32 ribosomal RNAs (rRNAs), 21 micro RNAs (miRNAs), 1 small RNA (sRNA), and 28 other types of ncRNAs. The snRNAs were classified as 30 spliceosomal RNAs (U2, U4 and U6), 1 minor spliceosomal RNA (U6atac), 3 C/D box small nucleolar RNAs (snoRNAs), and 3 H/ACA box snoRNA.

### Protein-coding gene annotation

MAKER v3.01.03 was employed with an integration of 3 strategies, including *ab initio* prediction, transcriptome-based and homology-based annotation^32^. The *ab initio* prediction was performed using BRAKER v2.1.5^33^, which automatically trained the predictors Augustus v3.3.4^34^ and GeneMark-ES/ET/EP 4.59_lic^35^, and made use of the mapped transcriptome data and protein homology information. The transcriptome information in BAM alignments was generated by HISAT2 v2.2.0^36^, and the protein sequences were extracted from the database OrthoDB10 v1^37^. For transcriptome-based annotation, RNA-seq data were firstly mapped to our assembly using HISAT2, and the transcriptome information in BAM alignments was produced. BRAKER was then run with the default parameters. With our reference assembly, transcriptome data were further assembled into transcripts using StringTie v2.1.4^38^. Protein sequences of three model insects (*Drosophila melanogaster*, *Bombyx mori* and *Tribolium castaneum*) and three representative species (*Daphnia magna*, *Apis mellifera* and *Rhopalosiphum maidis*) were downloaded from NCBI. Finally, MAKER was used to integrated the results of these three strategies using EVidenceModeler (EVM) pipeline v1.1.1^39^, weight 1, 2 and 8 was assigned to *ab initio*, protein homology and transcriptome, respectively. Overall, 14,906 protein-coding genes were predicted (Table 5), and the average gene length was 15,352.03 bp and the average CDS length was 1,569.20 bp. The average exon number of per gene was 9.45, with average exon length of 288.38 bp and average intron length of 1542.64 bp. On the basis of BUSCO analysis, 95.2% of the BUSCO database (insecta_odb10) genes were identified (single-copy genes: 85.3%, duplicated genes: 9.9%, fragmented genes: 1.0%, missing genes: 3.8%), further underlining the accuracy and completeness of gene predictions (Table 5).

Diamond v0.9.24 was firstly used to search the existing database UniProtKB with the sensitive model to obtain gene functions^40^. InterProScan v5.41–78.0^41^ was then used to screen proteins against the synthesis databases [Pfam, SMART, Gene3D, Superfamily, and Conserved Domain Database (CDD)] for predicting the protein domains. And eggNOG v5.0 database^42^ was searched for Gene Ontology (GO), Expression coherence (EC), Kyoto Encyclopedia of Genes and Genomes (KEGG) pathways, KEGG orthologous groups (KOs), and clusters of orthologous groups (COG) functional category annotation of the predicted protein-coding genes. Out of the protein-coding genes predicted in the pygmy grasshopper genome, 13,659 (91.63%) genes were matched the UniProtKB database (SwissProt + TrEMBL) to be assigned functions. Integrated analysis identified the homology and conserved protein domains for 13,011 (87.29%) genes. 11,178 genes were classified according to GO terms, and 9,968 genes were mapped to the KEGG pathway database. In addition, 8,711 KEGG ko terms, 2,972 Enzyme Codes, 11,393 Reactome pathways and 12,619 COG categories were predicted.

## Data Records

The genomic Illumina sequencing data were deposited in the NCBI Sequence Read Archive (SRA) database under accession No. SRR14826261^43^ and SRR14826262^44^.

The genomic Pacbio sequencing data were deposited in SRA database under accession No. SRR14843516^45^.

The transcriptome Illumina sequencing data were deposited in SRA database under accession No. SRR14825792^46^.

The Hi-C sequencing data were deposited in SRA database under accession No. SRR14827093^47^.

The assembled genome was deposited in the GenBank at NCBI under accession No. JAEMUL000000000^48^.

Genome annotation information of repeated sequences, gene structure and functional prediction is available in the Figshare database^49^.

## Technical Validation

The completeness and accuracy of the assembled genome were evaluated using two different strategies. First, BUSCO analysis showed that 97.4% (single-copied gene: 96.5%, duplicated gene: 0.9%) of 1,367 insect single-copy orthologues (in the insect_odb10 database) were successfully identified as complete, 0.6% were fragmented and 2.0% were missing in the assembly. Then, we mapped the sequencing data to the assembled genome for verifying the accuracy. The mapping rates was 94.92%, 93.62% and 96.77% for the Illumina, RNA-seq and PacBio data, respectively. Overall, the assessment results indicated that our *E. oculatus* genome assembly was complete, accuracy as well as high quality.

## Data Availability

No specific script was used in this work. The codes and pipelines used in data processing were all executed according to the manual and protocols of the corresponding bioinformatics software.
